# Cancer Patients’ Perceived Value of a Smartphone App to Enhance the Safety of Home-Based Chemotherapy: Feasibility Study

**DOI:** 10.2196/20636

**Published:** 2021-01-06

**Authors:** Nina Kongshaug, John-Arne Skolbekken, Arild Faxvaag, Eva Hofsli

**Affiliations:** 1 Cancer Clinic St. Olav's University Hospital Trondheim Norway; 2 Department of Clinical and Molecular Medicine Faculty of Medicine and Health Scienes Norwegian University of Science and Technology Trondheim Norway; 3 Department of Public Health and Nursing Faculty of Medicine and Health Sciences Norwegian University of Science and Technology Trondheim Norway; 4 Department of Neuromedicine and Movement Science Faculty of Medicine and Health Sciences Norwegian University of Science and Technology Trondheim Norway

**Keywords:** mhealth, mobile app, smartphone app, oral chemotherapy, patient safety, home-based cancer treatment

## Abstract

**Background:**

Oral anticancer therapies can be self-administered by patients outside the hospital setting, which poses challenges of adherence to a drug plan and monitoring of side effects. Modern information technology may be developed and implemented to address these pertinent issues.

**Objective:**

The aim of this study was to explore how a smartphone app developed through a stepwise, iterative process can help patients using oral chemotherapy to take their drug, and to report adherence and side effects in a reliable and verifiable manner.

**Methods:**

Fourteen patients starting capecitabine treatment were included in this study and used the smartphone app in addition to regular follow up of capecitabine treatment. Nine of these patients fulfilled the treatment plan and were interviewed based on a semistructured interview guide and the System Usability Scale (SUS). In addition, two focus groups were completed with 7 oncologists and 7 oncology nurses, respectively. Interview data were analyzed in accordance with the principles of systematic text condensation. Features of the app were also assessed.

**Results:**

The smartphone app provided the patients with a feeling of reassurance regarding correct adherence of their oral chemotherapy treatment. They used the app as a memory tool about their treatment and possible serious side effects, as well as for treatment education. Patients expressed concerns about using the app to report side effects that were not considered to be obviously serious, fearing overreporting. The health personnel expressed an overall positive attitude to integrate this new tool in their everyday work.

**Conclusions:**

Patients on oral chemotherapy treatment at home felt safe and found the app to be helpful. The app promoted learning about their treatment and made the patients more independent of the cancer clinic, reducing the need for the clinic’s limited resources for follow up of patients on oral anticancer medications.

## Introduction

Medical cancer treatment has changed from traditional intravenous chemotherapy given at hospitals to home-based oral anticancer therapies [1]. Oral chemotherapy can potentially be administered without the patient having to visit a cancer clinic given the advantages of avoiding the need for intravenous administration and its associated complications. Nevertheless, adherence is a widely known challenge for cancer patients on oral chemotherapy [2]. Given that patients will adhere to the drug plan and that side effects can be handled properly, such home-based therapy will make cancer patients more independent of the cancer clinic and also have potential to save costs [1,3]. Traditionally, the follow up of oral treatment adherence and side effects when patients are at home is done by a phone call to the patient and appointments at the clinic.

Vincent [4] defines patient safety as the management of risk over time to maximize benefit and minimize harm to patients in the health care system. Patient safety is an aspect of diagnostic services (eg, diagnostic safety [5]), therapy (eg, medication safety [6]), and health care coordination (eg, errors of commission and errors of omission [7]). Because patient safety is such an essential property of a health care system, health institutions are obliged to assess, monitor, and continuously work to improve the patient safety aspects of their services.

The context of this study is the safety of home-based therapy with capecitabine, an oral chemotherapeutic drug that is used in the treatment of gastrointestinal and breast cancers. Capecitabine has a wide dosage range and potential serious side effects such as diarrhea, hematologic toxicity, and hand-foot syndrome, although fatigue, nausea, and stomatitis are also frequently reported [8]. In recent years, the cancer clinic at St. Olav’s University Hospital in central Norway experienced hospital admissions due to diarrhea followed by acute renal failure as severe adverse events of capecitabine use, two of which were fatal. To improve the safety of home-based capecitabine treatment, the clinic immediately changed to a stricter dispensing and monitoring regimen, including the use of pill dispensers and follow-up phone calls. The clinic also developed a smartphone app to be used for reminding the patients to take the drug and to report side effects. This idea is also supported by a recent article describing that educating patients with timely medical information through their smartphones improves patient knowledge, treatment adherence, and clinical outcomes [9].

Approximately two-thirds of all people worldwide own a mobile phone [10]. This has created an ecosystem for mobile health (mHealth), the practice of medicine and public health supported by mobile devices [11]. Among their many prospects, mHealth apps offer the possibility for health care institutions to reach out to and interact with patients staying at home [10,12]. An mHealth taxonomy developed in 2015 described eight different use cases: point-of-care diagnostics, patient monitoring, wellness, compliance, education and reference, behavior modification, efficiency and productivity, and environmental monitoring [13]. There are examples of mHealth apps within oncology, including tools for point-of-care diagnostics (eg, melanoma diagnostic services) and tools for assessing patient-reported outcomes [14,15]. mHealth tools have been shown to increase medication adherence in patients with diabetes [16], but literature of their effects on adherence to oral anticancer therapies is lacking [10].

For an mHealth app to have an impact as a patient safety tool, it must be taken into use and perceived as useful by a majority of the patients in the target group [17]. We therefore sought to explore patients’ use of an app from an institutional perspective (ie, patient safety) as well as from the perspective of the patient (eg, perceived usefulness). The objective of this study was to explore how a smartphone app can assist patients in adhering to the capecitabine medication plan and for reporting side effects, and to also characterize the main features that the patients find to be most useful within the app.

## Methods

### Study Design

We performed a feasibility study with 14 cancer patients and 14 health care providers. Patients, physicians, and nurses were recruited at the cancer clinic of St. Olav’s University Hospital in central Norway in the period of March to October 2017. Nine of the 14 patients completed the test period and subsequently underwent a semistructured interview. The reasons for the 5 patients not completing the test period were as follows: capecitabine discontinued due to side effects (n=2), follow up by an oncologist outside St. Olav’s University Hospital (n=1), technical problems with downloading the app to the patient’s smartphone (n=1), and insufficient smartphone competence (n=1).

The oncology nurses assisted patients in downloading the app and setting up the treatment plan on their smartphones. In addition, two focus groups were completed with 7 oncologists and 7 oncology nurses, respectively. The main characteristics of the participants are summarized in Table 1.

**Table 1:** Demographic and treatment characteristics of the patients in this study (N=9).

Characteristic		Value
**Gender, n (%)**		
	Men	6 (67)
	Women	3 (33)
**Age (years), n (%)**		
	40-49	2 (22)
	50-59	4 (44)
	60-69	2 (22)
	70-79	1 (11)
**Oncology treatment plan, n (%)**		
	Chemoradiotherapy (radiation plus concomitant capecitabine)	6 (67)
	Intravenous chemotherapy every 3rd week plus capecitabine	3 (33)
**Smartphone system, n (%)**		
	IOS (iPhone)	6 (67)
	Android (Samsung)	3 (33)
**Level of education**		
	College/university	7 (78)
	High school	2 (22)

All patients were chemotherapy-naive, had a gastrointestinal cancer, and were indicated for capecitabine treatment. Inclusion criteria were patients >18 years of age with the ability to independently manage their medication and having good knowledge of how to use a smartphone (ie, used their phone for more than text messages and phone calls). The patients installed the app on their personal smartphone when they started the capecitabine treatment. All patients used the smartphone app in addition to regular follow up.

### Intervention

The smartphone app prototype was developed by a stepwise, iterative process in a multiprofessional group from St. Olav’s University Hospital and Norwegian University of Science and Technology (NTNU) in cooperation with information and technology communication system developers and designers, facilitated by the Technology Transfer Office of NTNU. The app is based on knowledge about capecitabine treatment and the current procedures for monitoring of these patients at the cancer clinic of St. Olav’s University Hospital. The source for the side effect component was Common Toxicity Criteria, version 4.03 [18]. Before starting the feasibility study, the prototype version of the app was tested on 10 colleagues to ensure acceptable usability.

**Figure 1:**
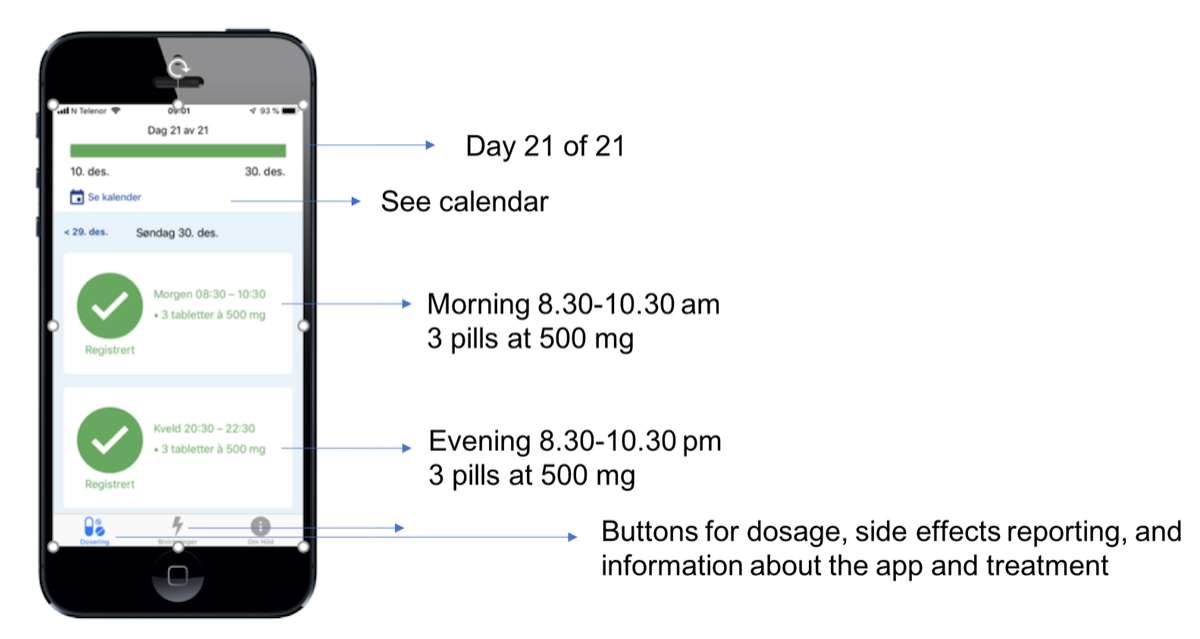
Features of the app for supporting medication adherence.

**Figure 2:**
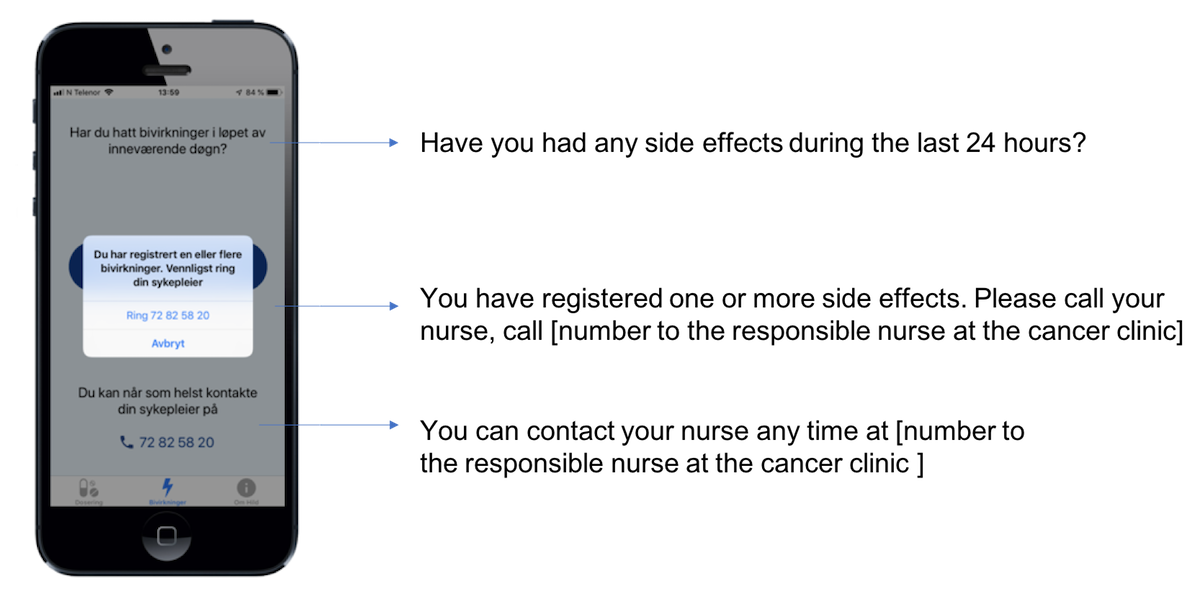
Features of the app for supporting management and reporting of side effects.

The app has two main features: (1) supporting adherence to the medication (Figure 1) and (2) management and reporting of side effects (Figure 2). The app alerts and reminds the patient to take the drug at the right time and offers a calendar visualization of the medication plan. The patients can register side effects, and the app provides a patient decision support system to call the nurse at the cancer clinic if needed. The prototype also provides a summary of all side effects registered in each capecitabine treatment cycle.

### Measures

Data were collected through semistructured interviews and with the System Usability Scale (SUS) as well as through two focus groups [19-21]. The SUS is considered to be an easy, quick, and reliable test of usability that is technology-agnostic, which is also available in a validated Norwegian translated version [20]. The questionnaire was not designed for statistical use in this study but was rather used as a starting point for the semistructured interviews.

### Data Collection

After 2 to 3 weeks on capecitabine treatment, a semistructured interview was performed with the patient. All patients were interviewed once. The interview guide focused on the patients’ experiences with the use of the app regarding correct capecitabine adherence and reporting of important side effects, experiences of safety of home-based chemotherapy treatment, and the possibility to obtain adequate help from health personnel when needed. The patients also provided information on a Norwegian validated version of the SUS questionnaire. This information was used as a starting point for the interviews. Patient interviews lasted from 12 to 25 minutes. The patient interviews took place at the hospital in an undisturbed room. The focus groups (with physicians and nurses) took place in a meeting room at the cancer clinic and lasted 60 minutes each. One of the authors acted as group moderator. An external researcher was co-moderator and took field notes. The focus group conversations covered the same topics as the patient interviews, but from a health personnel perspective. All interviews were digitally recorded and subsequently transcribed. Finally, the transcriptions were controlled against the recording.

### Data Analysis

Data were analyzed according to the principles of systematic text condensation [22]. This procedure consisted of four steps: (1) getting a total impression by reading all of the text materials and identifying preliminary themes; (2) identifying meaning units from both the technical aspects of the smartphone app and its use by patients, oncology physicians, and nurses; (3) abstracting condensates from each group and subgroup; and (4) creating synthesized descriptions of the patients’, oncology physicians’, and nurses’ experiences and opinions about the use of the smartphone app in the follow up of patients on capecitabine treatment. To some extent, we performed a stepwise analysis before completing data collection.

### Ethical Considerations and Approval

Participants provided informed consent based on oral and written information about the study and its purpose. The patients used the smartphone app as a supplement to regular follow up with pill dispensers and phone calls from the cancer clinic. All interviews were audiotaped and transcribed without any identifiable information so as to preserve the participants’ confidentiality. The soundtracks were deleted after transcription. Data stored on the patients’ smartphones were secured by a pin code. In this version of the app, the treatment plan was set up by a nurse on the patient’s personal phone, protected with a pin code unknown to the patient.

The Regional Committee for Medical and Health Research Ethics, South East, Norway confirmed that their approval was not required for this study (REK 2015/1581). The study was approved by the Norwegian Centre for Research Data and the Data Protection Officer for both the NTNU and St. Olav’s University Hospital.

## Results

### Overall Perspectives

The patients reported that the app aided them in adhering to the drug plan through reminders to take the drug and self-reporting of drug usage and side effects. The app also served as a memory aid, enabling them to learn more about the drug they were taking, and provided reassurance. Health personnel at the cancer clinic were concerned about the balance between making themselves more available to the patients and being able to handle the anticipated increase in the number of requests. When the study participants visited the outpatient clinic, nurses and physicians (with some exceptions) did not check the medication history via the app. Patients reported far fewer side effects than anticipated.

### Perceived Safety

One of the main findings of this study was that patients who used the app felt safer. Both the alerts on when to take the drug and which dose to take contributed to this feeling:

..you get an alarm on your mobile. You always have your mobile with you, it is a safety net. This was the greatest benefit with it.

Despite being instructed otherwise, some patients came to believe that the information they recorded on the app was shared with the clinic without delay. Unsurprisingly, the thought of having health personnel continuously monitoring their treatment and eventual side effects increased their sense of safety:

...so you feel that you are better followed-up [by the cancer clinic]. You know that if you register [the data], that someone will see it. It probably gives a better feeling of safety.

However, there was no such feature on the app version that the patients were testing. Hence, the use of the app made the patients believe they were being followed up more closely by the clinic than they actually were.

### Improved Memory and Interaction With the Clinic

The patients appreciated having access to a correct and always updated phone number to a nurse in the clinic:

Even if I have good control of my [information] sheets, it [the mobile app] is easier and more available. I had something to report about side effects, and they were there when you touched the screen, and then you get a phone number, and I got in touch with a nurse immediately… That helps a lot.

According to the nurse informants, this contrasted with previous patient reporting, where they spent whole days waiting for a nurse to call at day 3, 10, and 17 in the treatment cycle. Even if they did not have bothersome symptoms, they focused on the call at those specific days:

Many patients are at home all day, waiting for that phone call.

Patients that recorded their medication history on the app reported that they used these recordings to recount the details of their experiences with taking the drug:

For instance, when you are on chemotherapy, your memory is not as good as before you became ill, so it’s a benefit that you record if the side effects started on Tuesday or Thursday. It’s a nice aid… Because, there is a relation between the [treatment] doses, and then it’s easier to understand.

Hence, the app gave the patients an overview and a deeper insight into their side effect profile, which seemed to support a richer and more purposeful interaction with the clinic.

### Learning Promotion and Independence From the Clinic

According to the patients, the overview of serious side effects was always readily available on their mobile device, and it was quicker to open the app than having to find the information sheet provided by the cancer clinic. Some patients regularly used the app’s side effect component to assess their own side effects and decide whether to report the side effects to the clinic.

I have been into this side effect part [of the app] several times and assessed whether these are symptoms I have or not. So, I haven’t had side effects which should be reported [to the cancer clinic].

This finding was supported by the nurses who focused on the patients’ opportunity to act more independently while treated at home as the app assisted them in managing their own treatment and conceivable side effects. The clinicians also perceived the app as strengthening patients’ adherence to the right dosage and helping them to become more responsible for their own treatment.

### Suggestions for Improvement

The patients wanted to share information about adherence and side effects with their nurse and doctor:

I don’t know if this [app] will be connected to the electronic health record at the hospital or something like that? … Then I think it really can be useful, when the physicians can follow the adherence as well as the side effects. I think everything about surveillance and follow up is a good thing.

They also wanted an overview of all their hospital appointments integrated in the app, including receiving short messages if any of their appointments were changed:

But, there is something about the administration of the letters that we receive about appointments. They could have been dropped. Could have used the app instead. I think there are many opportunities here.

The patients expressed that they were ready for more digital communication than was available through this app and welcomed use of internet and smartphone tools for cancer treatment follow up.

### Reporting Side Effects

The physicians focused on the risk of information overload and how to filter what they needed to know and act on versus what not to engage in, given that the patients with the app could report on side effects whenever they wanted.

We walk around with [smart] watches and measurements of blood pressure… Why do we need all this information?… We need to have the information which impacts on the cancer treatment.

The nurses also emphasized the need for a good system for monitoring the patient registrations at the hospital, including when to act on them. However, they also focused on how the app could help patients take more responsibility for their cancer-related symptoms and treatment.

In its present design, the app did not allow for direct transfer of side effect reports. Instead, the app encouraged the patients to call the hospital whenever they experienced side effects that the clinic should be made aware of. However, patients were reluctant to use this function.

I had skin symptoms, then I saved and kept going to the next one, but then I got the message that said I should call my nurse, and... God, maybe I shouldn’t have done that?

As a result, patients reported far fewer side effects than anticipated, in contrast to the clinicians’ fear of information overload.

## Discussion

### Principal Findings

In this feasibility study, we have shown that cancer patients can use a smartphone app to be reminded to take a drug and report on their adherence to a cytostatic drug regimen in a reliable manner. Despite the fact that the app enabled reporting of side effects and offered side effect–specific advice, the app obviously failed to make patients comply with the hospitals’ guidelines for immediate reporting of serious side effects and adaptive adjustment of the therapeutic regimen.

Adherence is a known challenge for cancer patients on oral chemotherapy [2]. Patients regarded the drug-take reminding function useful and believed that it improved adherence. This observation is in line with those of previous studies that have explored the effects of drug-reminder apps in other clinical domains [23]. The drug-take reporting function of the app points toward a more comprehensive documentation of pharmaceutical interventions in oncology. However, whether the app actually increases adherence to the drug needs to be tested in a randomized clinical trial.

Patients using the app reported that they learned more about their treatment and that this made them less dependent on the cancer clinic. This might imply that an app can be an important supplement to the follow up by health care providers of cancer patients on oral anticancer treatment. This is in line with the results of Kessel et al [24] who showed that health-related quality of life reporting from oncological patients through a mobile app was accepted by patients.

An overall effect of the app was that it made the patients feel safer by working as a proxy for the clinic. The app offered the patients reassurance, assuming that they were very closely monitored by the cancer clinic despite being informed that the study version of the app did not have any feature allowing for automatic communication with the clinic. This effect was not intended and is an example of an unintended positive effect of health information technology [25]. In our study, all of the patients received standard follow up in addition to the app, and therefore there were no related ethical or patient safety issues. The next version of the app will be connected to the hospital network, enabling clinicians to follow up on patient-generated reports in a population health manner [26,27]. The ability of an app to provide reassurance to patients that suffer from a chronic, potentially life-threatening disease could increase patients’ adherence to the app and hence limit the well-known problem of user attrition [28,29]. This line of thought will be explored in future designs of the app.

In addition to objective parameters such as blood tests, correct reporting of side effects is a key for optimizing chemotherapy dosage [30-32]. Despite potential benefits, there are both technological and administrative challenges with integrating side effect reporting into practice [33]. We found that the side effect reporting function in the app served as a source of knowledge about side effects, but that the coupling between registering side effects and the following of rapid advice directly from the clinic often made the patients refrain from reporting. Taken together, the side effect reports failed to give a complete picture of what the patients were experiencing. This fear of reporting side effects could also be due to a fear of cessation of medication, possibly affecting their treatment negatively [34]. Motivated by the possibility of the side effect reporting, further work on the design of the side effect component of the app is needed, focusing on balancing the patients’ needs and understanding of reporting, as well as the health personnel’s needs to avoid information overload. With these aspects in mind, the new design of the side effect component of the app should allow the patients to register nuanced grading of side effects in which only specified severe side effects triggers an alert to the cancer clinic. Further work also includes a design change toward the clinicians’ need to find all patient data as a part of the electronic health record.

### Strengths and Limitations

Despite consistent findings in the patient interviews in this study, the small number of patients is a limitation to be overcome with future research. Another weakness is that the physicians, with a few exceptions, did not use the app in their daily work. This may be due to the fact that all of the data were stored on the patients’ private smartphones and that many of the physicians in a busy workday did not know who was included in the study and subsequently omitted to ask the patients.

The context of the use of this app differs from most mHealth apps that are oriented toward achieving wellness, as this is about illness and all potential dangers associated with having cancer and being exposed to risky therapies [35]. To our knowledge, this is the first study that indicates an app’s impact on the feeling of reassurance while using potentially toxic cancer medication. These results also provide a more complete picture of the adherence and side effects than we recently obtained with phone calls to the patients on specific days during the treatment schedule. 

### Conclusion

The growing number of new oral anticancer therapies encourages new thinking of the follow-up routines for this specific patient group. In conclusion, this app can be a helpful tool for supporting patients in the home-based part of their cancer treatment. The app must meet both patients’ and clinicians’ needs, but the patients’ and clinicians’ requirements for usefulness are not necessarily identical.

## Abbreviations

mHealth: mobile health
NTNU: Norwegian University of Science and Technology
SUS: System Usability Scale
